# Effects of pre-surgical administration of prostaglandin analogs on the outcome of trabeculectomy

**DOI:** 10.1371/journal.pone.0181550

**Published:** 2017-07-20

**Authors:** Takako Miki, Tomoko Naito, Miyuki Fujiwara, Ryoichi Araki, Rieko Kiyoi, Yusuke Shiode, Atsushi Fujiwara, Yuki Morizane, Fumio Shiraga

**Affiliations:** 1 Department of Ophthalmology, Okayama University Graduate School of Medicine, Okayama, Japan; 2 Japanese Red Cross Okayama Hospital, Okayama, Japan; Bascom Palmer Eye Institute, UNITED STATES

## Abstract

For primary open angle glaucoma (POAG), laser treatment or surgery is used when the target intraocular pressure (IOP) cannot be achieved by pharmacological agents, such as prostaglandin (PG) analogs; these drugs also have varied effects. We retrospectively reviewed the medical records of 74 POAG patients (74 eyes) whose IOP was inadequately controlled by PG analogs (bimatoprost [13 eyes], latanoprost [34 eyes], tafluprost [11 eyes], and travoprost [16 eyes]) and underwent primary trabeculectomy. The proportion of patients with no recurrent IOP elevation within 24 months post-trabeculectomy was significantly (P < 0.001) lower in the bimatoprost group (31.3%) than in the latanoprost (83.2%), tafluprost (45.5%), or travoprost groups (65.6%). Deepening of the upper eyelid sulcus (DUES) was observed before trabeculectomy in 18 of 74 eyes (24.3%) treated with bimatoprost (9 eyes; 50.0%), latanoprost (3 eyes; 16.7%), tafluprost (1 eye; 5.5%) and travoprost (5 eyes; 27.8%). The proportion of patients with no recurrent IOP elevation up to 24 months post-trabeculectomy was significantly (P < 0.0001) lower in the DUES(+) group (34.7%) than in the DUES(-) group (74.3%). Multivariate stepwise logistic regression analysis, with no recurrent IOP elevation used as dependent variable, and bimatoprost, latanoprost, travoprost, tafluprost, β-blocker, carbonic anhydrase inhibitor, brimonidine, gender, age, preoperative IOP, mean deviation, duration of PG analog use before surgery, and the number of ophthalmic solutions used as independent variables, identified only bimatoprost as a significant independent factor (P = 0.0368). Thus, the outcome of trabeculectomy varied depending on the PG analog used preoperatively, and bimatoprost use was associated with a high risk of recurrent IOP elevation up to 2 years post-trabeculectomy. This may indicate that the incidence of DUES differed with the PG analog used. Patients with glaucoma who are treated with bimatoprost should be monitored for DUES, and when these patients undergo trabeculectomy, the postoperative course of IOP should be followed carefully.

## Introduction

Currently, lowering intraocular pressure (IOP) is the only evidence-based, reliable treatment for glaucoma [1]. Various options are available as IOP-lowering therapy, including pharmacological agents, laser treatment, and surgery [2]. In the case of primary open angle glaucoma (POAG), laser treatment or surgery is generally considered when the target IOP is not achieved by pharmacological agents, or when pharmacological treatment cannot be conducted optimally due to adverse effects or poor compliance [3].

Prostaglandin (PG) analogs are used as first-line medications because of their excellent IOP-lowering effect, absence of systemic adverse effects, and requirement of few instillations [4]. In Japan, 4 PG analogs, i.e., bimatoprost, latanoprost, tafluprost and travoprost, are currently used clinically [5,6]. The known adverse effects specific to PG analogs include deepening of the upper eyelid sulcus (DUES), pigmentation of the eyelid and iris, and lengthening of the eye lashes [7–15].

Trabeculectomy is the surgery most commonly conducted for POAG. Trabeculectomy is a filtering surgery that involves removing a piece of limbal tissue beneath the scleral flap to create a new outflow for the aqueous humor. Combined use of anti-metabolites to inhibit scarring has greatly improved the surgical results of trabeculectomy. While IOP control is maintained long-term after trabeculectomy in most patients, there are cases in which IOP increases again, requiring repeat surgery.

In the present study, we focused on PG analogs that are the most frequently used treatment for POAG, and retrospectively analyzed the effects of various PG analogs used before trabeculectomy on the postoperative outcome. Upon finding a PG analog-dependent difference in postoperative outcome, we then focused on the development of DUES as a possible cause, and explored the relationship between the DUES status before trabeculectomy and the postoperative outcome.

## Methods

### Patients and subgroups

We retrospectively reviewed the medical records of POAG patients who underwent primary trabeculectomy at Okayama University Hospital between April 2012 and March 2015. This study was approved by the Ethical Committee of Okayama University (approval number: 1606–507). Informed consent was obtained from the subjects after a thorough explanation of the study objective and information collection was given in accordance with ethical principles based on the Helsinki Declaration. The study is registered with the UMIN Clinical Trial Registry (Trial Registration: UMIN000022926).

The diagnostic criteria for POAG were: (1) presence of open anterior chamber angle; (2) presence of glaucomatous optic disc change and associated glaucomatous visual field change; and (3) absence of ocular diseases except glaucoma or systemic diseases that may cause visual field disturbance. One eye of each patient was studied. When trabeculectomy was conducted in both eyes, the first eye that underwent trabeculectomy was included in the analysis. Patients who had a history of superior sclerocorneal incision cataract surgery or vitrectomy, and patients who had superior conjunctival scarring, were excluded from analysis.

All the trabeculectomies in this series were performed by one experienced surgeon (T.N.) using standardized procedures. Conventional trabeculectomy was performed with a fornix-based flap of the conjunctiva and Tenon’s capsule. A half- thickness 4 mm × 4 mm scleral flap was dissected to the clear cornea. A fluid-retaining sponge soaked with mitomycin (0.4 mg/mL) was applied to the superior sclera for 5 minutes, followed by washing with 100 ml of saline. After excision of the trabeculum, a peripheral iridectomy was performed. The scleral flap and conjunctiva were sutured firmly with 10–0 nylon. The conjunctiva was closed, and Seidel testing was performed at the conclusion of the procedure. Laser suturelysis was performed after surgery.

The PG analogs used immediately prior to trabeculectomy were bimatoprost 0.03% (Lumigan^®^ Ophthalmic Solution; Senju Pharmaceutical Co., Ltd., Osaka, Japan), latanoprost 0.005% (Xalatan^®^ Ophthalmic Solution; Pfizer Inc., Tokyo, Japan), tafluprost 0.0015% (Tapros^®^ Ophthalmic Solution; Santen Pharmaceutical Co. Ltd., Osaka, Japan), and travoprost 0.004% (Travatan Z^®^ Ophthalmic Solution; Alcon Japan, Ltd., Tokyo, Japan). Prostaglandin analogs prescribed by the referring doctors were not changed until surgery in all patients. The subjects were divided based on the PG analog used into the bimatoprost, latanoprost, tafluprost, and travoprost groups, and the post-trabeculectomy outcomes in the 4 groups were analyzed. Presence or absence of DUES was based on attending physicians’ evaluations and patients’ subjective symptoms. Three ophthalmologists who evaluated the upper eyelid photographs attained consensus on whether DUES was present in all patients. When all three observers agreed on the presence of obvious DUES and the patient also noticed signs of DUES, the patient was judged as DUES positive.

Patients with a description of DUES in the medical record prior to trabeculectomy were considered to be DUES-positive [DUES(+)]. The subjects were divided into a DUES(+) group and a DUES(-) group, and the postoperative outcome was compared between the 2 groups.

### Outcome measures

The primary outcome measure was the proportion of patients who demonstrated no recurrent IOP elevation up to 24 months after trabeculectomy in each PG analog group. Intraocular pressure was measured using a Goldmann applanation tonometer. Recurrent IOP elevation was diagnosed when 1 of the following criteria was fulfilled: (1) 2 or more consecutive episodes of IOP ≥ 15 mmHg were noted; (2) additional glaucoma ophthalmic solution was prescribed; (3) glaucoma surgery (excluding needling) was performed. Secondary outcome measures were the incidence of DUES in the PG analog groups, the relationship between the status of DUES and post-trabeculectomy outcome, and the factors associated with recurrent IOP elevation.

### Statistical analysis

Statistical analyses were conducted using JMP ver. 12.2 (SAS Institute Inc., Cary, NC, USA). The significance level was set at 5% for 2-tailed tests. Chi-squared tests and analysis of variance (ANOVA) were used to compare the patient background among the 4 groups. Cumulative proportions of patients with no recurrent IOP elevation in the various groups were calculated using the Kaplan–Meier method, and compared using the log-rank test. The incidence of DUES was compared among groups using univariate logistic regression analysis, and the odds ratio (OR), 95% confidence interval (CI), and P-value were calculated. In a multivariate logistic regression analysis, using stepwise selection, candidate independent variables (age, gender, preoperative IOP, preoperative mean deviation [MD], duration of PG analog use before surgery, frequency of preoperative ophthalmic solution instillation, preoperative bimatoprost use, preoperative latanoprost use, preoperative travoprost use, preoperative tafluprost use, preoperative β blocker use, preoperative carbonic anhydrase inhibitor use, and preoperative brimonidine use) were entered into the model.

## Results

### Patient background

A total of 74 patients (74 eyes) were analyzed (Table 1). They were categorized by the preoperative PG analog used into a bimatoprost group (13 patients, 13 eyes), latanoprost group (34 patients, 34 eyes), tafluprost group (11 patients, 11 eyes), and travoprost group (16 patients, 16 eyes). No significant differences in age and gender distribution were observed among the 4 groups (P = 0.2465 and 0.6020, respectively; chi-squared test and ANOVA, respectively). Preoperative IOPs were 18.2 ± 4.8 mmHg in the bimatoprost group, 20.8 ± 8.2 mmHg in the latanoprost group, 20.5 ± 5.7 mmHg in the tafluprost group, and 21.8 ± 8.4 mmHg in the travoprost group. Preoperative mean deviations (MD) were −16.9 ± 7.6 dB in the bimatoprost group, −19.5 ± 7.7 dB in the latanoprost group, −20.1 ± 6.7 dB in the tafluprost group, and −15.0 ± 7.0 dB in the travoprost group. No significant differences were observed in the preoperative IOP and preoperative MD among the 4 groups (P = 0.6067 and 0.1649, respectively; ANOVA). The duration of PG analog use before trabeculectomy differed among the 4 groups (P = 0.0038, ANOVA), because the various PG analogs were launched at different times. On the other hand, the number of concomitant glaucoma ophthalmic solutions used and the rate of concomitant use of brimonidine were significantly different among the 4 groups (P = 0.0043 and 0.0177, respectively; ANOVA and chi-squared test).

**Table 1:** Patient background of various prostaglandin (PG) analog groups.

		Bimatoprost group	Latanoprost group	Tafluprost group	Travoprost group	P value^b)^
		(13 patients, 13 eyes)	(34 patients, 34 eyes)	(11 patients, 11 eyes)	(16 patients, 16 eyes)	
Gender (n)	Male	5	24	7	10	0.2465
	Female	8	10	4	6	
Age (years)		69.9 ± 8.3	64.3 ± 16.1	67.5 ± 8.3	66.1 ± 11.9	0.6020
Preop IOP (mmHg)		18.2 ± 4.8	20.8 ± 8.2	20.5 ± 5.7	21.8 ± 8.4	0.6067
Preop MD (dB)		−16.9 ± 7.6	−19.5 ± 7.7	−20.1 ± 6.7	−15.0 ± 7.0	0.1649
Duration of PG analog use before surgery (months)		23.5 ± 11.0	63.0 ± 6.8	25.5 ± 12.0	33.4 ± 9.9	0.0038
No. of concomitant anti-glaucoma agents used^a)^		2.9 ± 0.2	2.4 ± 0.1	2.4 ± 0.2	2.9 ± 0.2	0.0043
β blocker n (%)		10 (76.9%)	30 (88.2%)	9 (81.8%)	15 (93.8%)	0.5655
Carbonic anhydrase inhibitor n (%)		11 (84.6%)	31 (91.2%)	10 (90.9%)	16 (100%)	0.4986
Brimonidine n (%)		9 (69.2%)	9 (26.5%)	4 (36.4%)	10 (62.5%)	0.0177

Data are expressed as mean ± standard deviation or number of patients (%).

^a)^Combination formulation is counted as a single agent.

^b)^Analyzed by chi-squared test and ANOVA. Preop = preoperative; IOP = intraocular pressure; MD = mean deviation

### Intraocular pressure after trabeculectomy

The IOP at 1 month after trabeculectomy was 8.3 ± 4.2 mmHg in the bimatoprost group, 8.2 ± 3.7 mmHg in the latanoprost group, 8.6 ± 4.1 mmHg in the tafluprost group, and 8.7 ± 3.9 mmHg in the travoprost group; there were no significant differences among the 4 groups (P = 0.9739, ANOVA).

Among the three criteria of recurrent IOP elevation, most patients (9 of 18) met the criterion of two or more consecutive IOPs ≥ 15 mmHg. No patients were started on additional glaucoma medications despite an IOP < 15 mmHg. Needling was performed in 1 patient in the latanoprost group and 1 patient in the bimatoprost group. Statistical analysis for intergroup difference was not possible due to the small number of cases.

**Fig 1:**
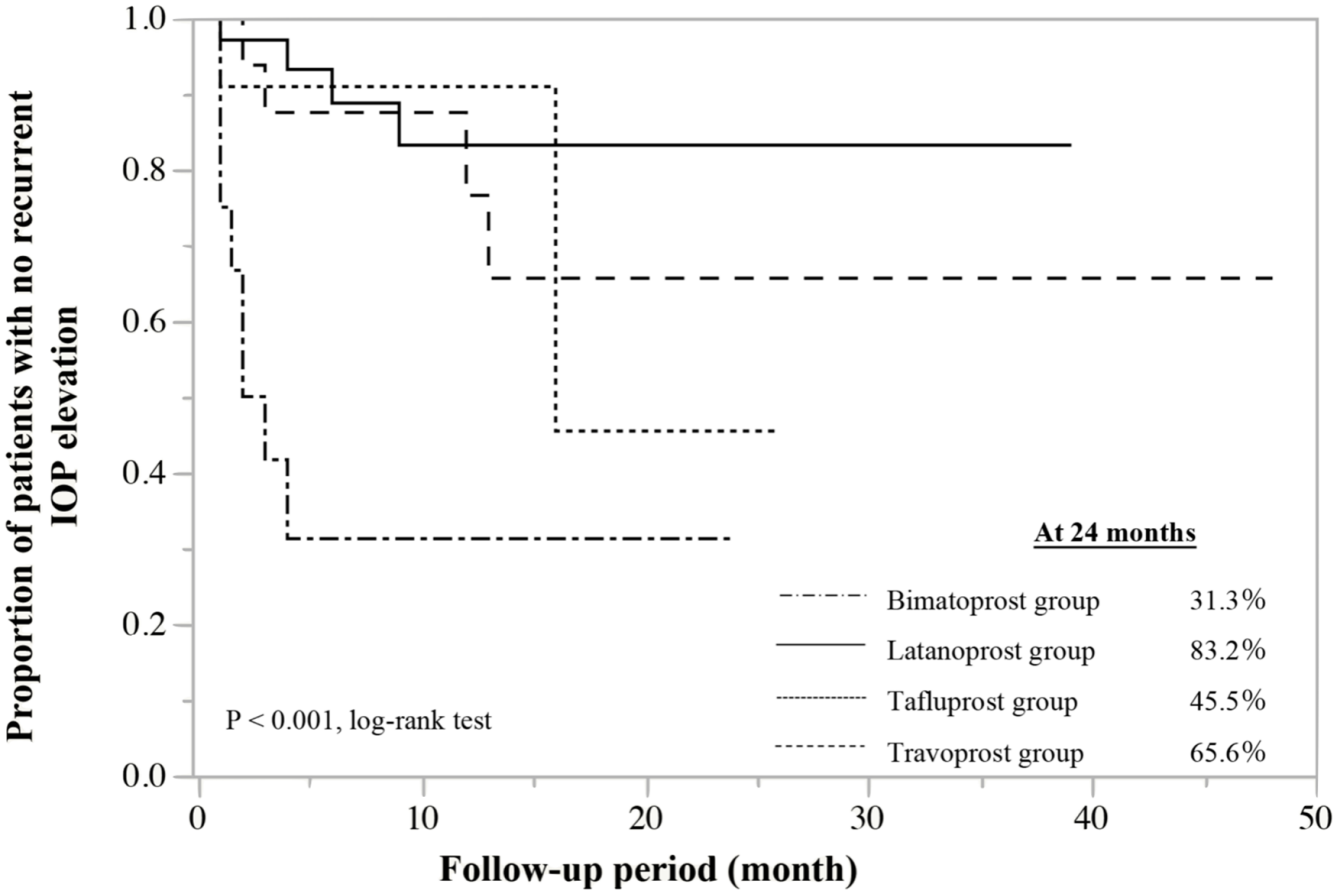
Cumulative proportions of patients with no recurrent intraocular pressure (IOP) elevation in various prostaglandin analog groups. The proportions of patients with no IOP elevation recurring up to 24 months post-trabeculectomy were 31.3% in the bimatoprost group, 83.2% in the latanoprost group, 45.5 in the tafluprost group, and 65.6% in the travoprost group.

As the primary outcome measure, the proportions of patients with no recurrent IOP elevation, up to 24 months post-trabeculectomy were calculated by Kaplan-Meier method; these were 31.3% in the bimatoprost group, 83.2% in the latanoprost group, 45.5% in the tafluprost group, and 65.6% in the travoprost group (Fig 1). A significant difference was observed in the recurrence rate of elevated IOP among the 4 groups (P < 0.001; log-rank test), and was the highest for bimatoprost among the PG analogs.

### Relationship with DUES

**Fig 2:**
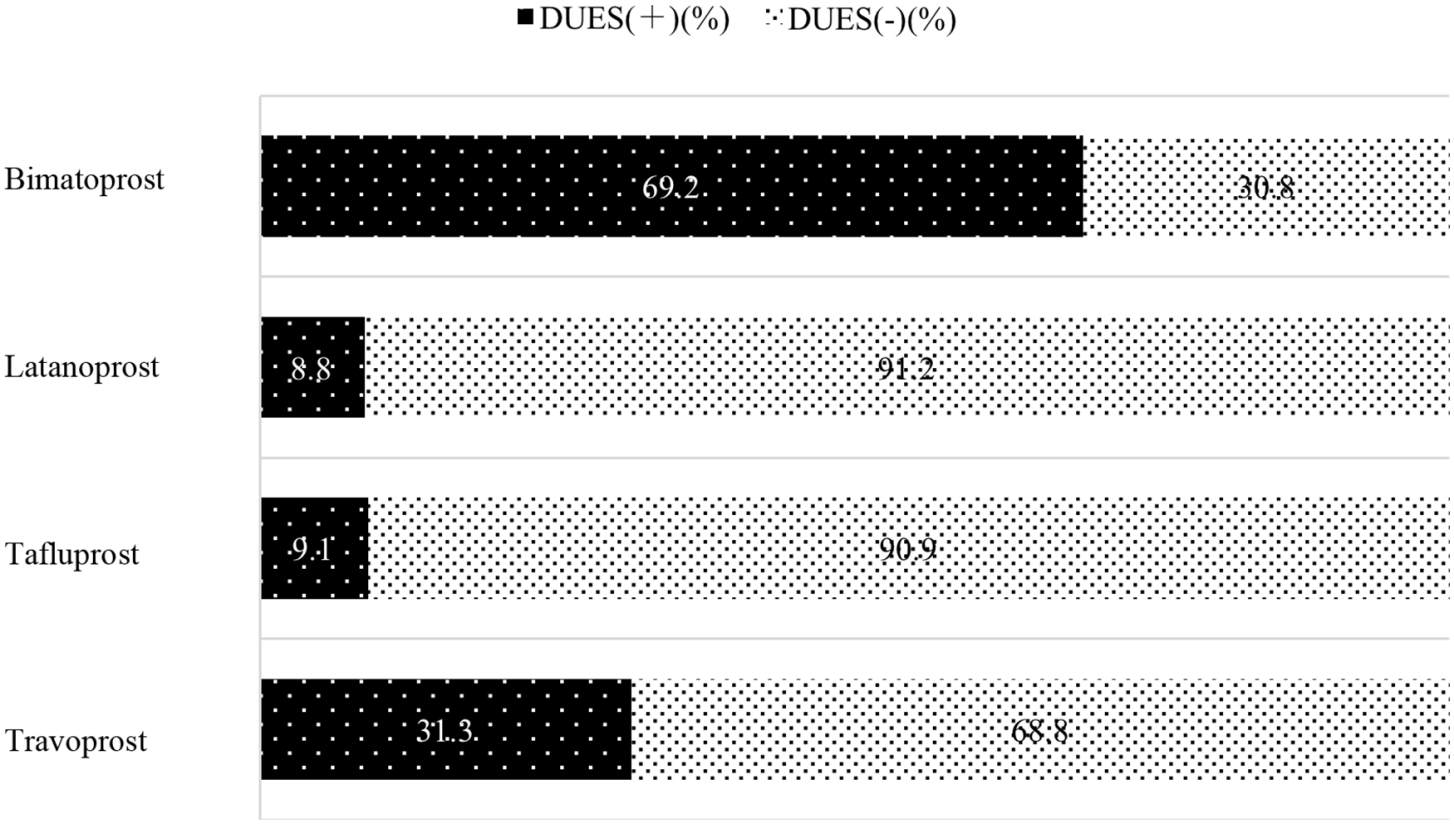
Incidence of deepening of the upper sulcus (DUES) in various prostaglandin (PG) analog groups. The incidence of DUES in various PG analog groups was 69.2% (9/13 eyes) in the bimatoprost group, 8.8% (3/34 eyes) in the latanoprost group, 9.1% (1/11 eyes) in the tafluprost group, and 31.3% (5/16 eyes) in the travoprost group, showing a high incidence in bimatoprost group compared to the other 3 groups.

The incidence of DUES in the various PG analog groups was 69.2% (9/13 eyes) in the bimatoprost group, 8.8% (3/34 eyes) in the latanoprost group, 9.1% (1/11 eyes) in the tafluprost group, and 31.3% (5/16 eyes) in the travoprost group, with the highest incidence in the bimatoprost group as compared to the other 3 groups (Fig 2). When the tafluprost group was used as reference, the OR for the development of DUES was not significantly different in the latanoprost group (OR: 0.97, 95% CI: 0.11 to 20.77; P = 0.9784) and the travoprost group (OR: 4.55, 95% CI: 0.59 to 95.16; P = 0.1545), but was significantly higher in the bimatoprost group (OR: 22.5, 95% CI: 2.89 to 492.85; P = 0.0017) (Table 2).

**Table 2:** Odds ratios for onset of deepening of the upper sulcus (DUES) in various prostaglandin (PG) analog groups.

	Odds ratio	95% Confidence interval	P value
**Tafluprost group**	1	-	-
**Latanoprost group**	0.97	0.11–20.77	0.9784
**Travoprost group**	4.55	0.59–95.16	0.1545
**Bimatoprost group**	22.5	2.89–492.85	0.0017

The 74 patients (74 eyes) analyzed were divided based on the presence of absence of DUES into a DUES(+) group (18 patients, 18 eyes) and a DUES(-) group (56 patients, 56 eyes). The 2 groups did not differ in terms of gender distribution and age (P = 0.9158 and 0.1491, respectively; chi-squared test and ANOVA). Preoperative IOPs were 18.4 ± 4.6 mmHg in the DUES(+) group and 21.1 ± 8.0 mmHg in the DUES(-) group, while preoperative MDs were −19.4 ± 7.3 dB in the DUES(+) group and −17.8 ± 7.6 dB in the DUES(-) group, with no significant differences in either parameter between the 2 groups (P = 0.1640 and 0.4355, respectively; ANOVA). On the other hand, the number of concomitant glaucoma ophthalmic solutions used and the rates of concomitant use of β blocker and carbonic anhydrase inhibitor were not significantly different between the 2 groups (P = 0.2694, 0.6528 and 0.6483, respectively; ANOVA and chi-squared test). However, the rate of concomitant use of brimonidine was significantly higher in the DUES(+) group (P = 0.0211; chi-squared test) (Table 3).

**Table 3:** Characteristics of patients in the DUES(+) and DUES(-) groups.

		DUES(+) group	DUES(-) group	P value^b)^
		(18 patients, 18 eyes)	(56 patients, 56 eyes)	
Gender (n)	Male	11	35	0.9158
	Female	7	21	
Age (years)		70.0 ± 13.1	64.9 ± 12.9	0.1491
Preop IOP (mmHg)		18.4 ± 4.6	21.1 ± 8.0	0.1640
Preop MD (dB)		−19.4 ± 7.3	−17.8 ± 7.6	0.4355
No. of anti-glaucoma agents used concomitantly^a)^		2.7 ± 0.6	2.5 ± 0.6	0.2694
β blocker n (%)		15(83.3%)	49(87.5%)	0.6528
Carbonic anhydrase inhibitor n (%)		17(94.4%)	51(91.1%)	0.6483
Brimonidine n (%)		12(66.7%)	20(35.7%)	0.0211

Data are expressed as mean ± standard deviation or number of patients (%).

^a)^Combination formulation is counted as one agent.

^b)^analyzed by unpaired *t*-test. Preop = preoperative; IOP = intraocular pressure; MD = mean deviation

**Fig 3:**
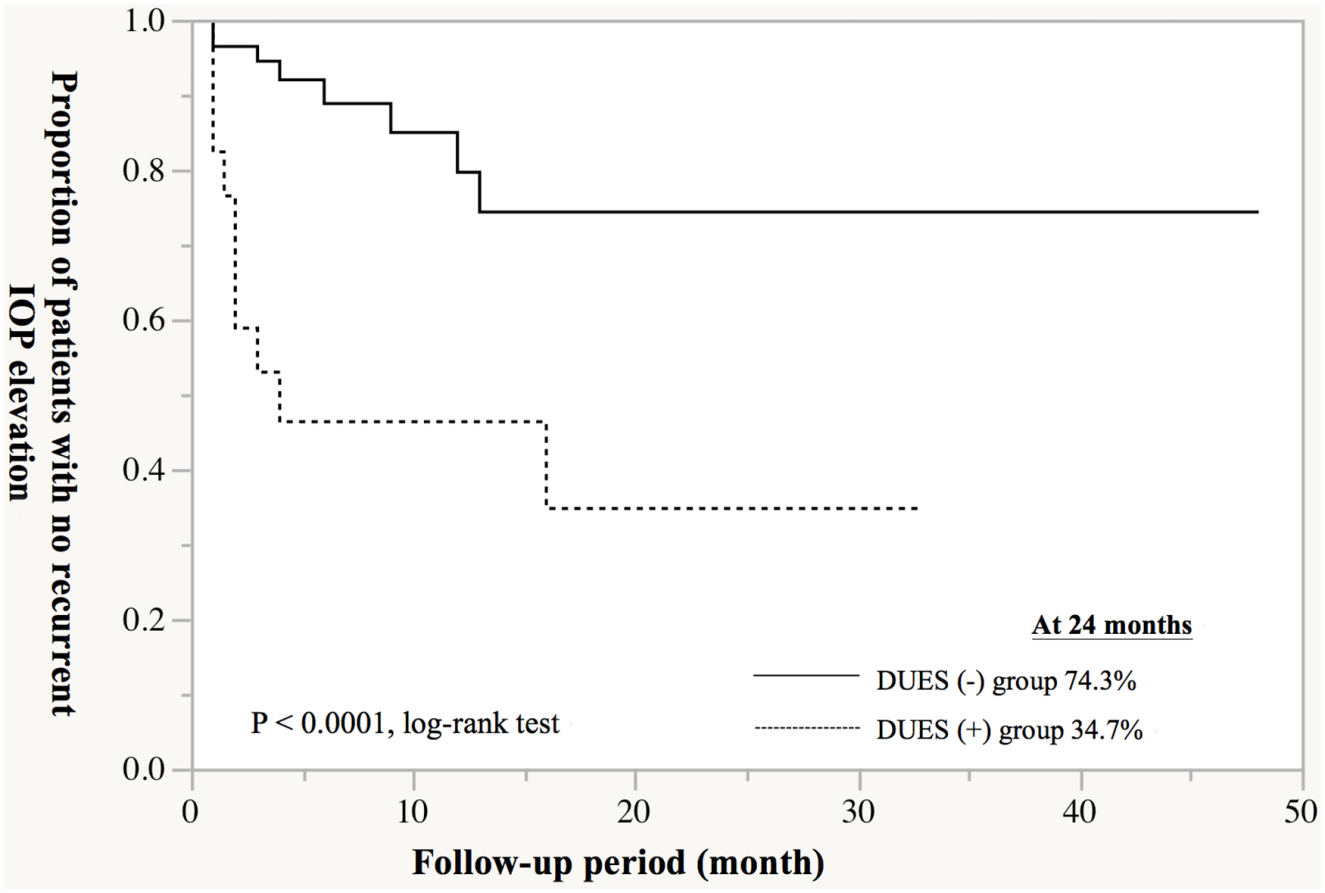
Cumulative proportion of patients with no recurrent intraocular pressure (IOP) elevation according to deepening of the upper sulcus (DUES). The proportion of patients with no recurrence of IOP elevation by up to 24 months after trabeculectomy was significantly lower in DUES(+) group than in DUES(-) group (34.7% vs. 74.3%; P < 0.0001, log-rank test).

**Fig 4:**
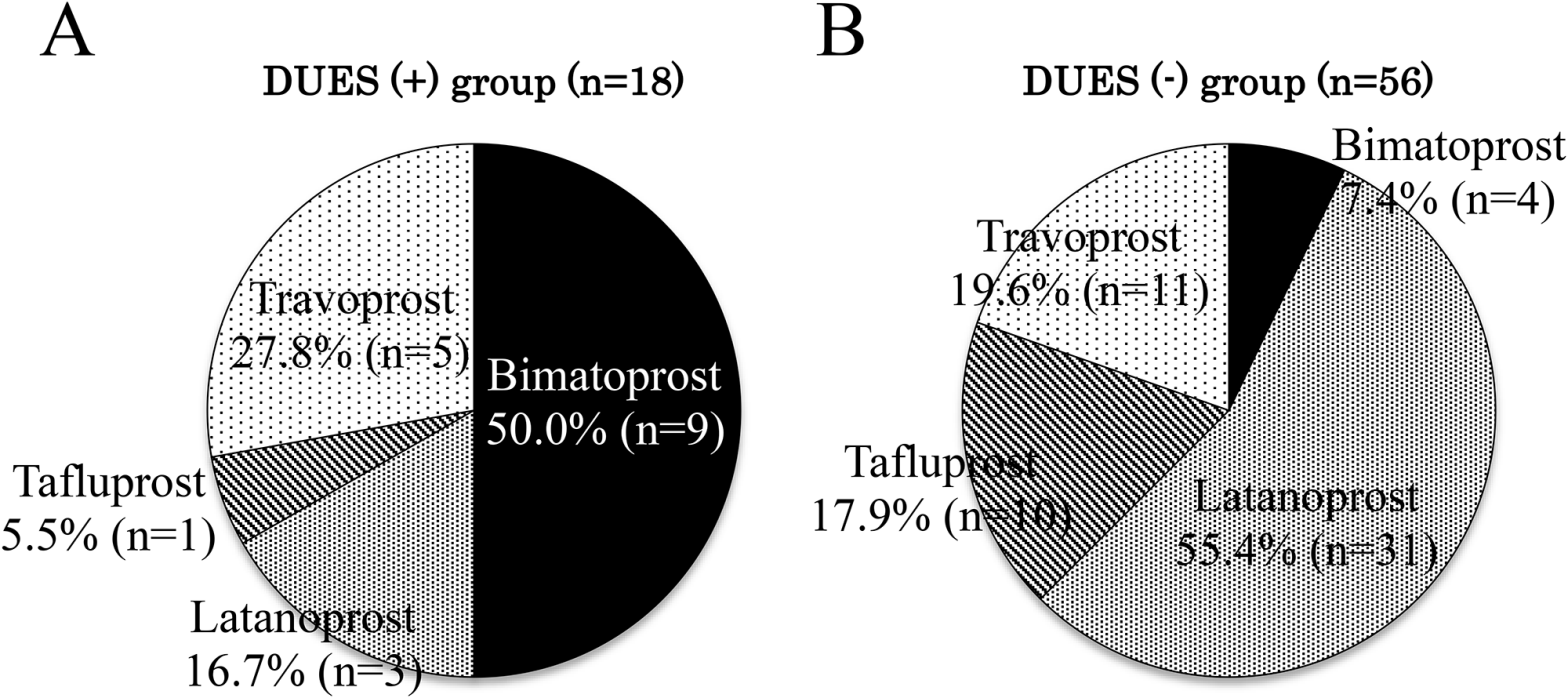
Ratios of prostaglandin (PG) analogs used before trabeculectomy in deepening of the upper sulcus DUES (+) and DUES (-) groups. When the PG analogs used prior to trabeculectomy were examined, the analog most frequently used in the DUES(+) group was bimatoprost (50.0%, 9/18 eyes), followed by travoprost (27.8%, 5/18 eyes), latanoprost (16.7%, 3/18 eyes), and tafluprost (5.5%, 1/18 eyes). The most frequently used analog in the DUES(-) group was latanoprost (55.4%, 31/56 eyes), followed by travoprost (19.6%, 11/56 eyes), tafluprost (17.9%, 10/56 eyes) and bimatoprost (7.1%, 4/56 eyes).

The proportion of patients with no recurrence of IOP elevation by up to 24 months after trabeculectomy was significantly lower in DUES(+) group than in DUES(-) group (34.7% vs. 74.3%; P < 0.0001, log-rank test) (Fig 3). When the PG analogs used prior to trabeculectomy were examined, the analog most frequently used in the DUES(+) group was bimatoprost (50.0%, 9/18 eyes), followed by travoprost (27.8%, 5/18 eyes), latanoprost (16.7%, 3/18 eyes), and tafluprost (5.6%, 1/18 eye) (Fig 4A). On the other hand, the most frequently used analog in the DUES(-) group was latanoprost (55.4%, 31/56 eyes), followed by travoprost (19.6%, 11/56 eyes), tafluprost (17.9%, 10/56 eyes) and bimatoprost (7.1%, 4/56 eyes) (Fig 4B).

### Factors associated with recurrent IOP elevation

Based on the above results, we conducted a multivariate analysis to identify the factors associated with recurrent IOP elevation up to 24 months after trabeculectomy. In a stepwise logistic regression analysis using no recurrent IOP elevation as the dependent variable and bimatoprost, latanoprost, travoprost, tafluprost, β blocker, carbonic anhydrase inhibitor, brimonidine, gender, age, preoperative IOP, preoperative MD, duration of PG analog use before surgery, and number of ophthalmic solutions used before surgery as independent variables, only bimatoprost was identified as the significant independent factor (P = 0.0368).

## Discussion

In the present series of POAG patients in whom IOP was inadequately controlled by PG analogs and who underwent trabeculectomy, the proportions of patients who experienced no recurrent IOP elevation constituted 83.2% in the latanoprost group, 65.6% in the travoprost group, 45.5% in the tafluprost group, and 31.3% in the bimatoprost group. These results demonstrated that postoperative outcome, as indicated by recurrent IOP elevation, differs depending on the PG analog used before trabeculectomy. In particular, the proportion of patients with recurrent IOP elevation was significantly higher among patients who used bimatoprost than in those who used the other 3 PG analogs. Furthermore, the results of multivariate analysis identified pre-trabeculectomy bimatoprost use as a significant risk factor for recurrent IOP elevation up to 24 months after trabeculectomy.

In terms of possible factors contributing to the difference in postoperative outcome depending on the PG analog used, we focused on DUES, which has been the focus of numerous reports in recent years. DUES is a specific adverse effect of PG analogs. In 2004, Peplinski et al. reported the first cases of DUES caused by bimatoprost therapy [7]. The mechanism of PG-induced DUES has been proposed to be as follows. Via activation of the prostanoid FP receptor, PG analogs decrease fat production in the orbital fat tissue; consequently, the orbital volume is reduced, deepening the eyelid sulcus [11, 16–21]. However, the incidence of DUES has been shown to differ depending on the type of PG analog. Inoue et al. reported that the incidence of DUES was 60.0% in patients using bimatoprost, 50.0% in those using travoprost, 24.0% in those using latanoprost, and 18.0% in those using tafluprost [22].

In the present study, the proportion of patients with IOP elevation recurring up to 24 months after trabeculectomy was significantly higher in patients who were positive for DUES than in those who were negative. Moreover, the DUES-positive rate was higher in patients who used bimatoprost (69%) than in those who used travoprost (31%), latanoprost (9%), or tafluprost (9%), suggesting that the high incidence of DUES in patients using bimatoprost may be associated with the unfavorable post-trabeculectomy outcome in these patients.

The present study has several limitations. First, Aihara et al. rated DUES by evaluating the photographs of the eyes and forehead. Before the initiation of treatment, PG was instilled in one eye [16]. Photographs were taken before treatment and every 2 months after starting treatment. The photographs were evaluated for the presence of DUES by 3 examiners, and a unanimous agreement was required for rating the eyes as DUES (+). Due to the retrospective design of our study, DUES was rated based on the attending physicians’ evaluations and patients’ subjective symptoms. However, the incidence in the present study was similar to that in a prospective evaluation study reported by Sakata et al. [23], indicating that the rating in our study was generally valid.

Next, besides DUES, several other factors may be responsible for the PG-related poor outcome after trabeculectomy, including conjunctival inflammation [24, 25]. Broadway et al. reported that long-term treatment with anti-glaucoma ophthalmic solutions induced conjunctival inflammation before surgery, causing fibroblast proliferation, as well as increases in macrophages, mast cells, and lymphocytes, in and beneath the conjunctival epithelium, which are risk factors for failure of trabeculectomy [26, 27]. However, conjunctival hyperemia is not a specific adverse effect of PG analogs. In addition, patients who undergo trabeculectomy generally have not achieved the target IOP despite the use of multiple anti-glaucoma medications. Since nearly all the subjects in the present study were also receiving multiple concomitant medications (100% in bimatoprost group, 97% in latanoprost group, 100% in tafluprost group, and 100% in travoprost group), it would seem invalid to simply compare conjunctival hyperemia in various PG analog groups, and concluding that conjunctival hyperemia is a PG-related poor prognostic factor of trabeculectomy. However, in a meta-analysis of 32 randomized controlled trials in patients with POAG and ocular hypertension, the risk of conjunctival hyperemia was higher for PG analogs compared to timolol, with relative risks (95% CI) of 4.66 (3.49–6.23) for bimatoprost, 2.30 (1.76–3.00) for latanoprost, 4.34 (2.34–8.04) for tafluprost, and 3.92 (3.04–5.05) for travoprost [28]. Therefore, the frequency of conjunctival hyperemia is generally high when PG analogs are used, and the possibility that conjunctival hyperemia is a risk factor for a poor outcome cannot be excluded.

Furthermore, eyelid hardening associated with prostaglandin-associated periorbitopathy (PAP) may also be a factor in poor prognosis. PAP is an adverse effect specific to PG analogs, and the term PAP describes a constellation of symptoms, including DUES, upper lid ptosis, involution of dermatochalasis, periorbital fat atrophy, mild enophthalmos, inferior scleral show, increased prominence of lid vessels, and tight eyelids [29, 30]. When eyelid hardening occurs due to PAP, the upper eyelid acts as a pressure eye patch and compresses the filtration bleb, affecting the formation and maintenance of the bleb. Further study is required to examine the effect of preoperative PAP caused by PG analogs on the outcome of trabeculectomy.

Baudouin et al. pointed out the possibility that benzalkonium chloride (BAK) may have deleterious effect on postoperative bleb function [31, 32]. However, in the present study, the rate of no recurrent IOP elevation was the highest in the group using latanoprost that has the highest BAK concentration. Therefore, the principal component is probably involved in maintaining bleb function. The preservatives used in the four prostaglandin analogs are as follows: 0.005% BAK in Lumigan^®^, 0.02% BAK in Xalatan^®^, 0.001% BAK in Tapros^®^, and SofZia^®^ in Travatan Z^®^.

Previous reports suggested that topical prostaglandin analogs did not affect the outcome of laser trabeculoplasty treatment [33,34]. On the other hand, we found that the outcome of trabeculectomy varied depending on the PG analog used before surgery. The difference in the effect of PG analogs on the two procedures may be due to the difference in level of PG exposure. Drug exposure at the conjunctiva with a bleb formed by trabeculectomy is vastly higher than that at the angle (inside the anterior chamber) that is irradiated by laser trabeculoplasty (approximately 0.01% of ophthalmic solution reaches the anterior chamber) [35–37].

## Conclusion

In the present study, we retrospectively analyzed the effect of PG analogs used prior to trabeculectomy on postoperative outcome in POAG patients with inadequate IOP control by PG analogs. In the present series, the outcome of trabeculectomy differed depending on the PG analog used before surgery, and the results suggested a high risk of recurrent IOP elevation up to 24 months after trabeculectomy in patients who used bimatoprost before surgery. The difference in incidence of DUES depending on the type of PG analog may be a factor. The sample size for individual PG groups was relatively small, especially for the bimatoprost group. Further large-scale study is required to confirm the findings.

The findings of the present study indicate that when performing trabeculectomy for patients with glaucoma, the status of DUES should be confirmed and the postoperative course of IOP should be followed carefully. In patients using bimatoprost but with poor IOP control, who are considering the option of surgical therapy in the future, medical treatment should be monitored to prevent the onset of DUES as far as possible. When trabeculectomy is performed in eyes that have already developed DUES, the postoperative course should be followed very carefully for recurrence of IOP.
